# World Health Organization Tool for Benchmarking Ethics Oversight of Health-Related Research with Human Participants

**DOI:** 10.31729/jnma.8839

**Published:** 2024-12-31

**Authors:** Namita Ghimire, Rojina Basnet, Richa Acharya, Santoshi Adhikari, Shashi Verma, Mohan Raj Sharma

**Affiliations:** 1 Nepal Health Research Council, Ramshah Path, Kathmandu, Nepal; 2 Department of Neurosurgery, Institute of Medicine, Tribhuvan University, Kathmandu, Nepal

**Keywords:** *ethical committee*, *health research*, *human participant*, *Nepal*, *WHO ethics benchmarking tool*

## Abstract

Benchmarking ethics oversight is a process of aligning comparing and evaluating the strengths and room for improvement of ethical review and approval process across different institutions or the country.

The World Health Organization tool for benchmarking ethics oversight is designed to help countries assess and improve their systems for ethically overseeing health-related research involving human participants. It identifies gaps in legal frameworks, committee structures, resources, accountability and transparency while promoting best practices and policy alignment. Nepal has made progress through the research regulations body like the Nepal Health Research Council , but challenges such as inconsistent implementation, oversight of the implementation and resource limitations remain. The tool offers an opportunity to strengthen Nepal's health research governance and regulation but it must be adapted to fit local contexts, emphasizing accountability, transparency, capacity-building, and improved communication.

## INTRODUCTION

Human research involves significant risks and can sometimes go wrong.^1^ Despite researchers' intention to help humanity, sometimes human beings may be harmed because of ethical insensitivity, neglect or disregard.^2^ Ethical oversight of human research is therefore essential in protecting the rights of research participants, enhancing research validity, and maintaining scientific or academic integrity.^3^

Ethical oversight of health research is grounded in a commitment to core principles that guide all health research designs and practices,^4^ including autonomy, beneficence and non-maleficence, voluntary participation, informed consent, privacy and confidentiality, risk-benefit assessment, and result communication.^5^

Considering the need for ethical oversight, World Health Organization (WHO) has developed a comprehensive tool that plays a crucial role in assessing the capacity of national governments, ethics committees, and research institutions to ensure adequate ethical oversight of health research involving human subjects. The tool helps to identify best practices and potential areas for improvement in upholding ethical standards by evaluating strengths, limitations, and existing policies and practices of ethics committees. Its goals are to ensure honesty, objectivity, integrity, carefulness, openness, transparency, accountability, social responsibility, non-discrimination, competence, and legality in the responsible conduct of research.

The WHO benchmarking tool for ethical oversight aids in the assessment of WHO Member States' capacity to oversee health-related research involving human subjects ethically. It highlights the advantages and disadvantages of research ethics-related policies, procedures, and frameworks and directs the formulation of suggestions to address the gaps.^6^ It also encourages keeping an eye on how these suggestions are being implemented. Beyond increasing capacity, the tool also supports public trust in health research, best practices, policy alignment, and participant safety in both routine and emergency public health scenarios. Ethical oversight of health research, rooted in the Belmont Report's principles, has been the cornerstone of protecting human participants in research.^5^

To date, most countries have established some sort of research ethics review system.^3^ Yet, despite the World Health Organization (WHO) standards for research ethics review existing since 2011, no extensive mechanisms are there to measure adherence to the standards. In an effort to fill this gap, WHO has developed a set of draft indicators for use in measuring the quality of research ethics review systems. The indicators were drafted with the input of a diverse group of international experts and stakeholders.

Nepal has made significant progress in establishing a research ethics framework through bodies like the Nepal Health Research Council (NHRC) and 60 Institutional Review Committees (IRCs).^7^ An important step toward encouraging responsible conduct in health research in Nepal has been taken with the establishment of the Ethical Review Board as an autonomous body which acts as the main body for examining and approving health research protocols.^8^ To guarantee the ethical and responsible conduct of research in Nepal, the NHRC has created extensive guidelines. Prior to its creation, there were no particular rules governing various kinds of research, including experiments involving animals. The first National Ethical Guidelines was introduced in 2001 which were later updated in 2011, 2019, and 2022. The National Guideline on Clinical Trials with Pharmaceutical Products (2005), the Guidelines for Institutional Review Committees (IRCs) (2005, 2016), and the Ethical Guidelines for the Care and Use of Animals in Health Research (2005) are further important guidelines. These rules have grown to be essential for directing moral research procedures throughout Nepal.^9^ Despite the global recognition of these ethical guidelines, there are no comprehensive systems in place to measure adherence to these standards. Also, challenges persist in ensuring that national ethics bodies like the NHRC consistently meet international standards across all areas, including transparency, accountability, and capacity-building. The WHO tool provides a structured approach for evaluating and improving these systems, aiming to ensure that ethical standards are upheld across all levels of health research governance.

## MAIN POINTS OR ARGUMENTS:

**Evaluating Nepal's Ethics Oversight System through the WHO Tool**

The workshop in Nepal offered an opportunity to apply the WHO benchmarking tool in assessing the country's current ethics oversight practices. During the sessions, stakeholders including ethics committee members and researchers identified the tool's potential to improve the efficiency and fairness of the ethical review process.

**Legal Provisions and Regulatory Framework**

One of the primary indicators in the tool is the presence of a robust legal framework that mandates ethical review for all health-related research involving human participants. Nepal's current legal requirements^10^ align with this indicator, but gaps remain in the oversight of the implementation and enforcement of these regulations, especially in terms of clinical trial implementation and ensuring that ethics committees are independently monitored. To monitor and regulated the IRCs, NHRC has establish the IRC accreditation sub-committee since 2019^11^ and monitoring of the approved study is being done in regular basis based on the type of risk involved in the study. As per the NHRC annual report only few percent of the implementation of the study is being monitored.^12^

**Structure and Composition of Ethics Committees**

Participants at the workshop emphasized the important of establishing a well-defined structure of ethics committees, along with the roles and responsibilities of the members. According to the NHRC National Ethical Guidelines 2022, ethics committee must ensure that members are selected based on diverse experts to uphold ethical standards in research. While the NHRC's Ethical Review Board (ERB) is commended for its robust framework and contributions to ethical review processes, there remains notable variability in IRCs concerning members expertise and adherence to procedural standards. Ensuring consistency across IRCs in line with National Ethical Guidelines and other relevant international code of conduct is essential for fostering a cohesive ethical oversight system.

**Resources and Capacity Building**

One of the key factors for effectively running an ethics committee is the institution's commitment to providing resources, both financial and human.

However, participants have highlighted that maintaining consistent allocations for both remains a major challenge. Many ethics committees rely on temporary staff, which undermines long-term capacity building. A crucial recommendation is to establish permanent staff positions with ongoing training and sufficient resources to address this gap and ensure the sustainable functioning of the ethics committees.

**Transparency and Accountability**

Transparency was identified as a key area for improvement. While Nepal has taken steps to make guidelines and lists of registered protocols publicly accessible, the extent of information shared remains inconsistent. The workshop emphasized the need to make the ethical approval processes more transparent, while still safeguarding the confidentiality of researchers' ideas.

**Challenges in ethical approval process**

There was a clear dedication of Nepal's ethics committee for improving ethical oversight of ethics committee as well as the implementation of the health research. The collaborative spirit among ethics committees, researchers, head of the institutions and international experts was inspiring. However, the discussions also revealed deep-rooted challenges, particularly the bureaucratic delays and lack of communication between researchers and ethics Committees. A representative from researchers in Nepal expressed they have experienced the frustration of navigating complex review and approval processes. Hence, implementing the WHO tool could help streamline these procedures and foster better dialogue between all stakeholders involved.

**Counterarguments**

One possible criticism of the WHO tool is that it may impose a one-size-fits-all approach to ethics oversight, failing to account for the unique cultural and institutional contexts of countries like Nepal. While the tool provides a comprehensive framework, some participants argued that local adaptability should be emphasized to avoid rigid applications that may not address country-specific needs. Concerns were raised about the practicality of the tool's indicators in underresourced settings, where capacity constraints may hinder full compliance.

## CONCLUSIONS

The WHO tool for benchmarking ethics oversight provides Nepal with a valuable opportunity to enhance its health research regulations and governance systems. By identifying strengths and areas for improvement, it can help increase transparency, accountability and ensure that ethics committees are well-resourced, ultimately aligning Nepal with global standards for ethical research. However, to be truly effective, the tool must be adapted to Nepal's specific context, culture and capacities. As we move forward, fostering open communication between head of the institutions, researchers and ethics committees promoting equitable and regular capacity-building, and improving resource allocation will be essential steps in fully realizing the tool's potential for advancing ethical research in the country.
